# Association between added sugars and frailty in U.S. adults: a cross-sectional study from the National Health and Nutrition Examination Survey 2007–2018

**DOI:** 10.3389/fpubh.2024.1403409

**Published:** 2024-06-12

**Authors:** Jian Ji, Jie Qiu, Yijing Tao, Ming Xu, Bin Pei, Chaoshen Wu, Guoxin Huang, Da Qian

**Affiliations:** ^1^Intensive Care Unit, Nanjing Drum Tower Hospital Group Suqian Hospital, Suqian, China; ^2^Department of Thyroid and Breast Surgery, Shaoxing People's Hospital, Shaoxing, China; ^3^Department of Cardiology, Changshu Hospital Affiliated to Soochow University, Changshu No.1 People's Hospital, Changshu, China; ^4^Department of Burn and Plastic Surgery-Hand Surgery, Changshu Hospital Affiliated to Soochow University, Changshu No.1 People's Hospital, Changshu, China; ^5^Department of Evidence-Based Medicine Center, Xiangyang No.1 People’s Hospital, Hubei University of Medicine, Xiangyang, China; ^6^Central Laboratory, Changshu Hospital Affiliated to Soochow University, Changshu No.1 People's Hospital, Changshu, China

**Keywords:** adult, added sugars, frailty, NHANES, nutrition, cross-sectional study

## Abstract

**Objective:**

There are various detrimental effects of excessive added sugar consumption on health, but the association of added sugars with frailty remains elusive. We aimed to examine the association between added sugar intake and frailty among American adults in the present cross-sectional study.

**Methods:**

This cross-sectional study is based on the National Health and Nutrition Examination Survey (NHANES) database. Data from NHANES spanning from 2007 to 2018 on frailty, added sugars, and covariates were collected. Added sugars were categorized into quartiles according to the recommended percentages by institutions. Weighted multivariable logistic regression was used to analyze the relationship between frailty and added sugars. Subgroup analysis was conducted based on sex, age, body mass index (BMI), smoking, alcohol consumption, hypertension, and diabetes status.

**Results:**

This study included 16,381 participants, with 13,352 (81.51%) in the non-frailty group and 3,029 (18.49%) in the frailty group. We found that added sugars were positively associated with frailty, and subgroup analysis showed that participants who were male, over the age of 60, had a low BMI, had previously smoked and consumed alcohol, had no hypertension, or had diabetes mellitus (DM) were more likely to be frail. Added sugar intake was positively associated with frailty. Subgroup analysis showed that the association was strongest in males, those aged >60, those with a low BMI, former smokers, former alcohol consumers, and people with no hypertension or DM. When added sugars are classified by energy percentage, populations with more than 25% of their energy coming from added sugars have similar results, with a higher prevalence of frailty.

**Conclusion:**

Added sugars are positively associated with a higher risk of frailty, and the association is stable among different populations.

## Introduction

Frailty, a complex clinical condition characterized by diminished physiological capacity, can make the human body more vulnerable to external stressors (1–3). With the challenge of population aging globally, the prevalence of frailty escalates rapidly, accompanied by a substantial surge in healthcare costs related to frailty. Moreover, frailty will increase the risk of adverse outcomes, including falls, fractures, chronic diseases, cancers, and mortality (4).

Added sugars are defined as sweeteners added to food during processing or preparation, excluding naturally occurring sugars present in vegetables, fruits, and milk (5). Sugary drinks such as energy drinks, juice, and soda are the main sources of added sugars (6). The National Health and Nutrition Examination Survey (NHANES) investigates a spectrum of added sugar sources, including brown sugar, sugarcane syrup, corn syrup, honey, molasses, and white sugar (7, 8). Previous studies have demonstrated the association of added sugars with elevated risks of obesity (9, 10), diabetes mellitus (DM), and cardiovascular disease (CVD) (11), which are intrinsically correlated with frailty. Despite previous studies focusing on factors precipitating frailty in the older adults, there is limited evidence revealing the association between added sugars and frailty in the general population.

Considering the negative impact of added sugars on health, the American Heart Association (AHA) recommended that the average daily intake of added sugars for males be no more than 150 kcal and for females be no more than 100 kcal (12). The 2020 Dietary Guidelines for Americans (DGA) advocates for stringent limits on added sugar intake, emphasizing daily thresholds of up to 10% of total calories per day for added sugars (13). The World Health Organization (WHO) also recommends below 5% of total energy intake for added sugars (14). Investigators also recommend that people control their sugar intake not only for its physical health repercussions but also for its potential psychological implications. A lot of research indicate that added sugars not only increase the risk of various chronic diseases but also affect mental health, closely correlating with the occurrence of psychological issues (15). This may be associated with long-term high sugar intake damaging the neurotransmitter systems in the brain, such as the dopamine and serotonin systems, thereby impacting emotional regulation and mental wellbeing (16). To explore the association of added sugars with frailty in adults of different ages, we conducted a large-scale cross-sectional study based on the NHANES survey.

## Materials and methods

### Study population

NHANES is a nationwide survey conducted by the Centers for Disease Control and Prevention to examine the health and nutrition status of adult residents in the United States (US). Researchers from all over the world can access the official NHANES database to carry out various investigations. This cross-sectional study also utilized data from the NHANES 2007–2018 database (http://cdc.gov/nchs/nhanes, last accessed on 8 January 2024). NHANES group uses stratified and multi-stage sampling methods to collect representative demographical and clinical data through interviews, standard exams, and biospecimen collection (17). Detailed investigation projects and designs can also be found on the NHANES website. All participants have written informed consent, and the National Center approved the project for Health Statistics Research Ethics Review Board.

### Added sugars assessment

NHANES staff utilize 24-h dietary recall to assess the intake of added sugars. All NHANES participants were eligible for two 24-h dietary recall interviews, the first conducted at the mobile examination center and the second via phone. Dietary data (including total energy and added sugars) were derived from the US Department of Agriculture Pyramid Equivalent Database/Food Pattern Equivalent Database (MPED/FPED) files, based on total nutrient intake over two consecutive days. We directly extract dietary data derived from dietary interview recalls from the NHANES database. Considering that individual dietary expenditure is closely related to body size, metabolic efficiency, and physical activity, we used the percentage of added sugars energy (added sugars daily energy divided by total daily energy) for all analyses. The percentage of added sugars energy intake was categorized through two methods: the first method is based on quartiles of intake, and the second method is to divide the percentages into <5, 5–10, 10–25, and ≥ 25 based on cutoff values recommended by various institutions. In addition, in accordance with NHANES analysis guidelines, the first and second dietary data were averaged in the present study.

### Frailty assessment

The frailty index (FI) in this study was derived from 46 items in the NHANES database, excluding factors related to dietary intake or nutritional status, such as difficulty using fork and knife, difficulty preparing meals, glycohemoglobin, triglyceride, creatinine, hemoglobin, mean corpuscular volume, total cholesterol, glucose, and sodium (18, 19). The FI, ranging from 0 to 1, signifies the susceptibility to frailty, with higher scores indicating higher risk. In this study, we divided the FI score into increments of 0.1, with a cutoff point of 0.2 to categorize frailty.

### Covariates

Demographical and clinical data were obtained at the NHANES website and utilized as covariates, including sex (male and female), age, body mass index (BMI), race (white, Mexican, black, and other), education level (under high school, high school or equivalent, above high school), marital status (married, living with a partner, separated, divorced, widowed, never married), poverty, lifestyles such as smoking (never, former, now), alcohol use (never, former, mild, moderate, heavy), physical activity, underlying diseases such as hypertension (yes or no), DM (yes or no), stroke (yes or no), CVD (yes or no), and cancer (yes or no).

### Statistical analysis

All analyses in the present study were followed by an NHANES statistical tutorial. Weighted analysis was used according to multi-stage probability sampling methods in NHANES. To yield integer values without changing the distribution, the FI was multiplied by 100. The variable wtmec2y is used to weigh NHANES sample data to represent the population of the US. This weight is calculated based on NHANES’ multi-stage sampling design and complex sampling methods. In this study, if the continuous variable follows the normal distribution, it is presented in the form of mean and standard deviation (SD). In the comparison between groups, T-test is adopted. If the continuous variable does not conform to the normal distribution, it is shown as mean and quartile, and non-parametric tests were used for comparison between groups. Categorical variables were presented as percentages, and chi-square tests were used to compare groups. NHANES has a complex multi-stage sampling design; hence, during statistical analysis, we weighted the samples and obtained weights for each individual to extend the sample distribution to the entire U.S. population, reflecting the characteristics of the overall population. In logistic regression analysis, we applied the sample weighting coefficients provided by NHANES and adjusted for potential confounders related to frailty to ensure the representativeness and generalizability of the results. This study used three models: non-adjusted weighted logistic regression; adjustments for age, gender, and race/ethnicity; and adjustments for all covariates. Subgroup analysis was conducted stratified by sex (male/female), age (≦60 years, >60 years), BMI (<25, 25–30, >30), alcohol use (never, former, heavy, mild, moderate), smoking (never, former, now), diabetes (no, yes), and hypertension (no, yes). A two-tailed *p*-value of <0.05 was considered statistically significant.

## Results

### Baseline demographical and clinical characteristics

**Figure 1:**
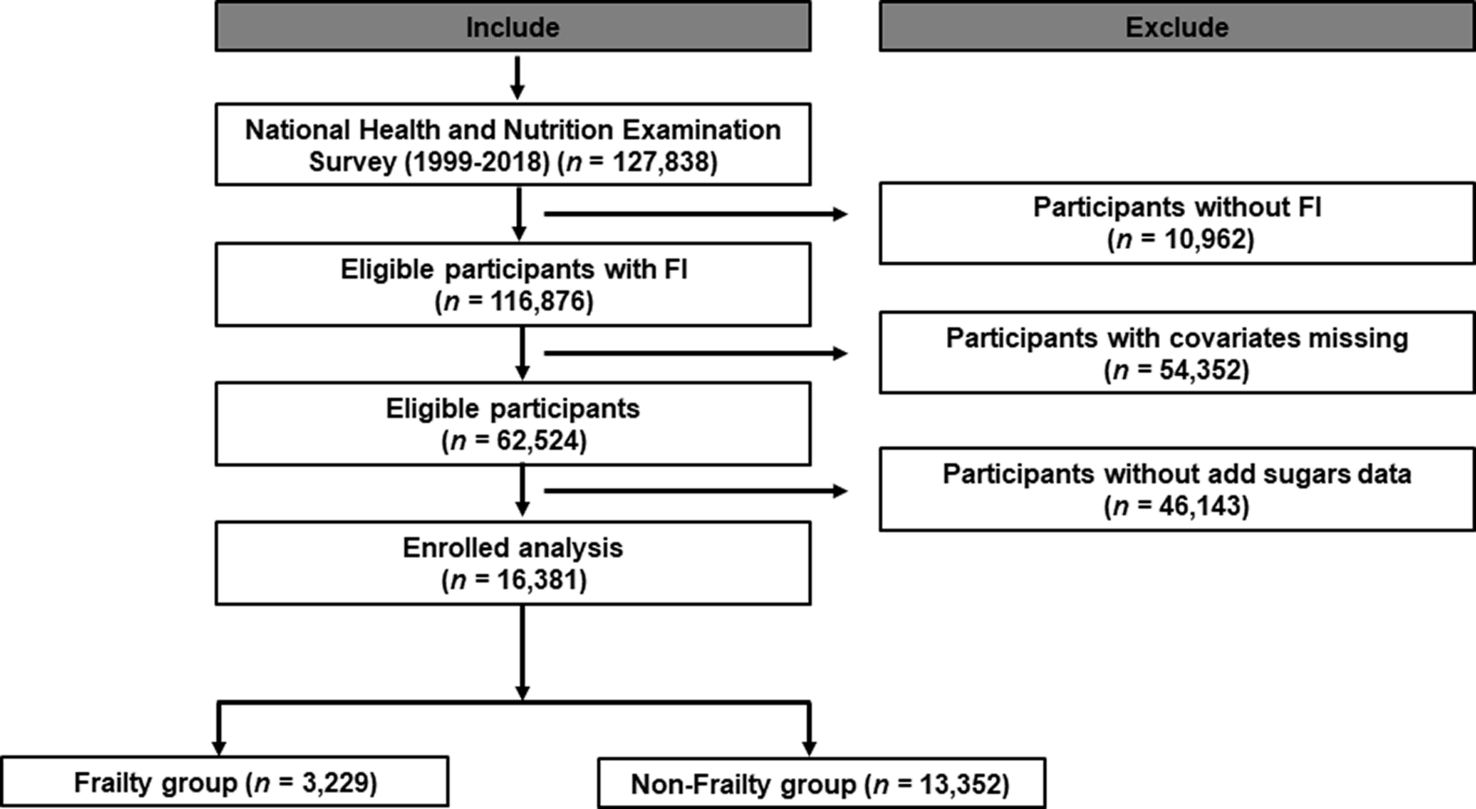
Study design overview. *Demography: including information about sex, age, BMI, race/ethnicity, education, marital status, and poverty.

Figure 1 describes a detailed study of population enrollment flow according to the inclusion and exclusion criteria. Based on the added sugars intake of all participants enrolled, we divided all participants into four groups on average (Q1: 0–7.11 kcal; Q2: 7.11–13.22 kcal; Q3: 13.22–22.18 kcal; and Q4: 22.18–162.90 kcal). In Table 1, baseline demographical and clinical characteristics of the study population are shown. A total of 116,876 participants were assessed for frailty in NHANES from 1999 to 2018, of which we had access to added sugars data for 82,541 participants. After excluding participants with missing covariates, a total of 49,811 participants were included. We eventually enrolled 16,381 participants from NHANES 2007–2018. As shown in Table 1, 13,352 (81.51%) participants were in the no-frailty group, and 3,029 (18.49%) participants were in the frailty group, representing 152,355,040 participants in the weighted analysis. A total of 51.69% of the study population were males and 48.31% were females. The weighted mean age of the study population was 45.83 years old, the mean BMI was 28.72 kg/m^2^, the mean poverty ratio was 3.13, and the median vitamin D intake was 3.55 mg.

**Table 1:** Weighted selected characteristics of the study population in females and males grouped added sugars intake quartiles, NHANES (weighted N = 152,355,040).

Character	Total	Q1	Q2	Q3	Q4	*p*-value
Sex, %						< 0.0001
Male	51.69	43.76	44.37	52.67	65.26	
Female	48.31	56.24	55.63	47.33	34.74	
Age, mean (SD)	45.83 (0.30)	47.46 (0.51)	47.77 (0.49)	45.98 (0.45)	42.30 (0.36)	< 0.0001
BMI, mean (SD)	28.72 (0.11)	28.71 (0.18)	28.53 (0.16)	28.67 (0.15)	28.94 (0.16)	0.3
Race/Ethnicity, %						< 0.0001
White	69.74	69.36	69.85	69.46	70.27	
Mexican	7.82	7.39	8.04	8.25	7.57	
Black	9.96	7.63	8.35	10.98	12.70	
Other	12.48	15.62	13.76	11.30	9.46	
Education, %						< 0.0001
Under high school	11.65	11.49	9.75	10.28	15.00	
High school or equivalent	21.79	18.45	19.59	22.16	26.71	
Above high school	66.56	70.06	70.66	67.56	58.30	
Marital status, %						< 0.0001
Married	54.98	57.68	57.08	55.44	49.94	
Living with partner	8.14	6.61	7.24	8.30	10.27	
Separated	2.09	1.91	1.73	2.32	2.36	
Divorced	10.11	9.37	10.47	9.68	10.87	
Widowed	4.13	5.36	5.00	4.10	2.17	
Never married	20.56	19.07	18.48	20.15	24.39	
Poverty, mean (SD)	3.13 (0.04)	3.30 (0.05)	3.30 (0.06)	3.14 (0.06)	2.80 (0.06)	< 0.0001
Smoking, %						< 0.0001
Never	56.75	58.56	60.02	58.33	50.32	
Former	24.48	27.37	26.42	23.92	20.43	
Now	18.77	14.07	13.56	17.75	29.25	
Alcohol intake, %						< 0.0001
Never	9.53	10.49	8.99	9.87	8.83	
Former	11.01	9.73	9.89	10.58	13.71	
Mild	38.22	36.24	41.85	39.06	35.74	
Moderate	18.70	22.08	20.21	18.29	14.47	
Heavy	22.54	21.46	19.06	22.20	27.25	
Physical activity, mean (SD)	2,360 (900, 5,880)	2,160 (840, 4,920)	1920 (780, 4,800)	2,160 (900, 5,640)	3,360 (1,080, 8,640)	< 0.0001
Hypertension, %						< 0.001
No	66.18	62.45	65.07	67.80	69.14	
Yes	33.82	37.55	34.93	32.20	30.86	
Diabetes, %						< 0.0001
No	91.57	87.17	90.16	93.75	94.91	
Yes	8.43	12.83	9.84	6.25	5.09	
Stroke, %						0.37
No	97.90	97.86	97.72	98.38	97.66	
Yes	2.10	2.14	2.28	1.62	2.34	
CVD						0.08
No	93.16	92.35	92.68	93.36	94.20	
Yes	6.84	7.65	7.32	6.64	5.80	
Cancer						0.03
No	90.11	89.46	88.66	90.37	91.86	
Yes	9.89	10.54	11.34	9.63	8.14	
Vitamin D, median (IQR)	3.55 (1.90,6.10)	3.10 (1.55,5.20)	3.45 (1.90,5.90)	3.95 (2.15,6.50)	3.85 (1.95,6.95)	< 0.0001
Frailty score, %						0.18
No	85.27	85.15	84.74	86.77	84.40	
Yes	14.73	14.85	15.26	13.23	15.60	

### Association between added sugars and frailty

Logistic regression analysis showed a positive association between added sugars and frailty in models 1, 2, and 3. As the percentage contribution of added sugars to energy supply increased by 1%, the OR was 1.00 (95%CI: 1.00 to 1.01; *p* = 0.54), 1.01 (95%CI: 1.01 to 1.02; *p* < 0.001), and 1.01 (95%CI: 1.00 to 1.01; *p* = 0.02), respectively. According to the quartiles of added sugars, all three models in Table 2 also indicated a positive correlation between added sugars percentage and frailty. Compared to the participants in the first quartile, participants in the fourth quartile had a higher prevalence of frailty compared with those in the fourth quartile in both partially adjusted model 2 (*p* < 0.001; OR = 1.47, 95%CI: 1.24 to 1.75) and fully adjusted model 3 (*p* < 0.001; OR = 1.42, 95%CI: 1.18 to 1.70). Furthermore, we found that among individuals with added sugars energy intake exceeding 25%, the incidence of frailty significantly increased in both models 2 and 3, with an OR of 1.41 (95%CI: 1.13 to 1.76) and 1.42 (95%CI: 1.11 to 1.80), respectively.

**Table 2:** Weighted ORs (95%CIs) of the associations between added sugars and frailty.

Exposure	Model 1^a^	*p*-value	Model 2^b^	*p*-value	Model 3^c^	*p*-value
Added sugars (%kcal)	1.00 (1.00, 1.01)	0.54	**1.01 (1.01, 1.02)**	<0.001	**1.01 (1.00, 1.01)**	0.02
Quartile of %kcal added sugars						
Q1	Ref	Ref	Ref	Ref	Ref	Ref
Q2	1.03 (0.85, 1.25)	0.74	1.03 (0.85, 1.24)	0.79	1.18 (0.92, 1.50)	0.18
Q3	0.87 (0.72, 1.06)	0.17	0.97 (0.80, 1.16)	0.71	1.12 (0.90, 1.40)	0.3
Q4	1.06 (0.92, 1.22)	0.43	**1.47 (1.24, 1.75)**	<0.001	**1.42 (1.18, 1.70)**	<0.001
Percentage of energy from added sugars						
<5	Ref	Ref	Ref	Ref	Ref	Ref
5–10	0.95 (0.76, 1.18)	0.95	0.93 (0.73, 1.19)	0.58	1.09 (0.82, 1.46)	0.55
10–25	0.89 (0.73, 1.08)	0.89	0.97 (0.79, 1.18)	0.74	1.19 (0.92, 1.53)	0.19
>25	1.00 (0.82, 1.21)	1	**1.41 (1.13, 1.76)**	0.003	**1.42 (1.11, 1.80)**	0.01

a Model 1: added sugars.

b Model 2: added sugars, age, sex, and race/ethnicity.

c Model 3: all the covariates.Bold value: the value has statistical significance.

### Subgroup analysis

Table 3 shows the subgroup analysis results of the association of added sugars with frailty among different populations, indicating that the association between added sugars and frailty was more obvious among males (OR = 1.01; 95%CI: 1.00 to 1.02), individuals aged >60 (OR = 1.02; 95%CI: 1.01 to 1.04), individuals with low BMI (OR = 1.01; 95%CI: 1.00 to 1.02), former smokers (OR = 1.02; 95%CI: 1.01 to 1.04), former alcohol consumers (OR = 1.02; 95%CI: 1.00 to 1.03), individuals without hypertension (OR = 1.01; 95%CI: 1.00 to 1.02), and individuals with DM (OR = 1.02; 95%CI: 1.00 to 1.04) (as the percentage contribution of added sugars to energy supply increases by 1%). Individuals in the fourth quartile compared to those in the first quartile, males (OR = 1.63; 95%CI: 1.17 to 2.25), individuals aged >60 years (OR = 2.45; 95%CI: 1.81 to 3.33), individuals with low BMI (OR = 1.91; 95%CI: 1.21 to 2.99), former smokers (OR = 1.83; 95%CI: 1.23 to 2.72), former alcohol consumers (OR = 2.29; 95%CI: 1.43 to 3.67), and former alcohol consumers with mild level of now alcohol intake (OR = 1.50; 95%CI: 1.06 to 2.14), individuals with DM (OR = 2.39; 95%CI: 1.34 to 4.27), and individuals without hypertension (OR = 1.41; 95%CI: 1.13 to 1.76) were more likely to have frailty. In addition, we classified results based on the percentage of energy from added sugars, and found that males (OR = 1.85; 95%CI: 1.19 to 2.85), individuals aged >60 years (OR = 3.35; 95%CI: 2.24 to 5.03), individuals with low BMI (OR = 2.12; 95%CI: 1.25 to 3.60), former smokers (OR = 2.29; 95%CI: 1.38 to 3.80), former alcohol consumers (OR = 2.54; 95%CI: 1.37 to 4.68), individuals with hypertension (OR = 1.51; 95%CI: 1.12 to 2.04), and individuals with DM (OR = 2.63; 95%CI: 1.36 to 5.08) were more likely to tend to have frailty.

**Table 3:** Stratified logistic regression analysis.

Character	Added sugars (%kcal)	Quartile of %kcal added sugars				Percentage of energy from added sugars			
		Q1	Q2	Q3	Q4	<5	5–10	10–25	>25
Sex									
Male	**1.01 (1.00, 1.02)**	Ref	1.12 (0.78, 1.60)	1.03 (0.71, 1.50)	**1.63 (1.17, 2.25)**	Ref	1.42 (0.94, 2.15)	1.38 (0.89, 2.13)	**1.85 (1.19, 2.85)**
Female	1.00 (0.99, 1.02)	Ref	1.19 (0.89, 1.58)	1.19 (0.89, 1.59)	1.19 (0.87, 1.64)	Ref	0.91 (0.66, 1.27)	1.06 (0.79, 1.42)	1.14 (0.79, 1.65)
Age									
= < 60	1.00 (1.00, 1.01)	Ref	1.11 (0.82, 1.48)	0.97 (0.72, 1.30)	1.09 (0.86, 1.38)	Ref	0.95 (0.66, 1.35)	0.95 (0.70, 1.29)	0.98 (0.73, 1.32)
>60	**1.02 (1.01, 1.04)**	Ref	**1.34 (1.00, 1.80)**	**1.45 (1.03, 2.03)**	**2.45 (1.81, 3.33)**	Ref	**1.50 (1.06, 2.11)**	**1.83 (1.27, 2.63)**	**3.35 (2.24, 5.03)**
BMI									
Normal	1.01 (1.00, 1.02)	Ref	1.19 (0.87, 1.64)	1.20 (0.84, 1.71)	1.31 (0.91, 1.88)	Ref	1.55 (0.97, 2.50)	1.48 (0.97, 2.25)	**1.63 (1.01, 2.63)**
Low	**1.01 (1.00, 1.02)**	Ref	1.48 (0.99, 2.24)	**1.54 (1.02, 2.32)**	**1.91 (1.21, 2.99)**	Ref	1.46 (0.90, 2.37)	**1.70 (1.09, 2.67)**	**2.12 (1.25, 3.60)**
High	1.01 (1.00, 1.01)	Ref	1.11 (0.72, 1.71)	0.99 (0.71, 1.37)	**1.36 (1.04, 1.78)**	Ref	0.77 (0.50, 1.19)	0.93 (0.62, 1.37)	1.14 (0.82, 1.60)
Smoke									
Never	1.01 (1.00, 1.02)	Ref	**1.46 (1.00, 2.12)**	1.21 (0.84, 1.75)	**1.53 (1.12, 2.08)**	Ref	1.20 (0.79, 1.82)	1.30 (0.85, 1.98)	**1.44 (1.01, 2.05)**
Former	**1.02 (1.01, 1.04)**	Ref	0.99 (0.70, 1.41)	0.93 (0.63, 1.36)	**1.83 (1.23, 2.72)**	Ref	1.04 (0.62, 1.74)	1.10 (0.71, 1.69)	**2.29 (1.38, 3.80)**
Now	1.00 (0.99, 1.01)	Ref	0.90 (0.57, 1.44)	1.13 (0.69, 1.85)	0.93 (0.58, 1.48)	Ref	0.97 (0.56, 1.70)	1.01 (0.58, 1.76)	0.84 (0.48, 1.48)
Alcohol intake									
Never	1.02 (1.00, 1.03)	Ref	1.06 (0.61, 1.86)	1.28 (0.77, 2.12)	**2.20 (1.12, 4.33)**	Ref	1.07 (0.60, 1.89)	1.21 (0.68, 2.15)	**2.24 (1.05, 4.78)**
Former	**1.02 (1.00, 1.03)**	Ref	1.34 (0.88, 2.04)	**1.71 (1.08, 2.72)**	**2.29 (1.43, 3.67)**	Ref	1.48 (0.83, 2.64)	**1.72 (1.03, 2.87)**	**2.54 (1.37, 4.68)**
Mild	1.01 (1.00, 1.02)	Ref	1.19 (0.79, 1.77)	1.27 (0.85, 1.89)	**1.50 (1.06, 2.14)**	Ref	0.98 (0.60, 1.59)	1.15 (0.70, 1.91)	1.38 (0.87, 2.19)
Moderate	1.01 (0.99, 1.03)	Ref	0.86 (0.52, 1.43)	0.90 (0.56, 1.44)	1.19 (0.68, 2.11)	Ref	0.65 (0.33, 1.29)	1.04 (0.65, 1.69)	1.20 (0.64, 2.26)
Heavy	1.00 (0.98, 1.02)	Ref	1.34 (0.85, 2.10)	0.74 (0.51, 1.09)	0.94 (0.54, 1.63)	Ref	1.55 (0.92, 2.63)	0.99 (0.61, 1.62)	1.00 (0.47, 2.13)
Hypertension									
No	**1.01 (1.00, 1.02)**	Ref	1.24 (0.93, 1.64)	1.08 (0.79, 1.48)	**1.35 (1.00, 1.82)**	Ref	0.97 (0.65, 1.43)	1.02 (0.77, 1.37)	1.25 (0.89, 1.76)
Yes	1.01 (1.00, 1.01)	Ref	1.12 (0.84, 1.50)	1.12 (0.84, 1.49)	**1.41 (1.13, 1.76)**	Ref	1.18 (0.83, 1.69)	1.28 (0.91, 1.78)	**1.51 (1.12, 2.04)**
DM									
No	1.01 (1.00, 1.01)	Ref	1.16 (0.90, 1.48)	1.07 (0.84, 1.38)	**1.30 (1.04, 1.62)**	Ref	0.94 (0.68, 1.31)	1.07 (0.80, 1.44)	1.24 (0.94, 1.64)
Yes	**1.02 (1.00, 1.04)**	Ref	1.20 (0.76, 1.88)	1.22 (0.72, 2.09)	**2.39 (1.34, 4.27)**	Ref	1.57 (0.98, 2.52)	1.45 (0.88, 2.36)	**2.63 (1.36, 5.08)**

Bold value: the value has statistical significance.

## Discussion

The adverse effects of added sugars in food on the human body have been widely studied. In this study, we investigated the correlation between added sugars and frailty by including a large, multi-ethnic study population from the NHANES public database. Our research indicates a positive correlation between added sugars and frailty, and this association is stable across different populations. This finding may provide references and assistance for the prevention and treatment of frailty, especially in the older adults population.

Previous studies indicate that a higher consumption of added sugars is associated with unhealthy dietary habits (20), increasing the risk of DM, CVDs, and obesity (21). Consequently, various institutions have proposed recommendations for the maximum allowable intake of added sugars. For instance, the UK Nutrition Science Advisory Committee advises that added sugars should not exceed 10% of total daily caloric intake, with the ultimate goal of reducing sugar consumption to 5% of calories or lower (22). In addition, the AHA has established even stricter guidelines regarding daily added sugar intake for adults (12). Previous studies have also revealed that excessive added sugar consumption can lead to nutrient deficiencies among the older adults (23), as foods and beverages rich in added sugars tend to be high in empty calories and lacking in essential micronutrients. In some literature, the food sources of added sugars were divided into three categories: treats, toppings, and sugar-sweetened beverages (SSBs) (24); and other reported food sources of sugar can be divided into milk-based desserts, dairy products, sugary cereals, cakes and pastries, sugary products, fruit, and sugary drinks (8). It is crucial to recognize that different food sources result in different health outcomes. Moreover, with the continuous improvement of economic levels and quality of life, the intake of added sugars shows an increasing trend in lifestyles. However, the association between added sugars and frailty has not been investigated yet. Added sugars can affect physiological function and increase energy burden, which may contribute to limitations in physical activity and decreased function (21). Consequently, individuals with elevated added sugar intake may be at greater risk of experiencing frailty and displaying higher FI scores. In previous studies, researchers have already used the NHANES database to investigate frailty and its association with various health outcomes. Huang et al. investigated the relationship between FI and osteoarthritis (OA) and found that the incidence of frailty in OA patients is higher (25). Chen et al. also utilized the NHANES database to conduct a cross-sectional study and found FI was not only associated with chronic heart failure but also connected with all-cause mortality and cardiovascular mortality (26). Another study aimed to investigate the association between oral health and frailty in older adults Americans. The results of this study showed that oral health in the older population has a significant impact on the prevalence of frailty, whereas nutrient intake seems to have little impact on this association, emphasizing the importance of maintaining good oral health among the older adults (27). Additionally, researchers have also investigated the risk factors contributing to frailty based on the NHANES database. Sun et al. found that an anti-inflammatory diet can alleviate the adverse effects of poor sleep quality on frailty by investigating the diet and sleep quality of 9,007 participants from NHANES (3). Jayanama et al. investigated the association between an individual’s nutrient intake and mortality rates at different levels of frailty and found that higher alcohol consumption and higher levels of serum alpha-carotene, beta-carotene, beta-cryptoxanthin, total cholesterol, and LDL-c were associated with lower FI scores, while only low vitamin D was associated with increased mortality risk across all frailty levels (18). Another study found that being overweight or obese is associated with higher frailty levels. Surprisingly, overweight is a protective factor for mortality in moderately/severely frail people, and obesity grade 1 may be protective for mortality for people with at least a mild level of frailty. In contrast, obesity grades 2 and 3 may be associated with a higher mortality risk in the non-frailty population (2). Actually, before this study, some researchers were exploring the relationship between sugar intake and frailty. A prospective study conducted in Spain in 2017 indicated that among older adults people aged over 60, sugar intake might lead to frailty. However, the study included a relatively small number of participants, and the racial diversity was also limited (28). Furthermore, no research has been conducted on the relationship between added sugar intake and frailty in a larger general population. Controversially, in another prospective study, researchers also included 1,822 older adults individuals aged over 60 to primarily investigate the relationship between protein intake and frailty. However, in the analysis, the researchers simultaneously found no correlation between sugar intake and the risk of frailty (29). In addition, many researchers have studied the relationship between dietary patterns and frailty. Researchers have found that a more diverse diet and healthy eating habits may reduce the risk of frailty. However, in these studies, the addition of sugar has not been considered a significant indicator affecting frailty (30–32).

This cross-sectional study explored the association between added sugars and frailty by including 11 study cycles of NHANES. We found that a higher percentage of added sugar intake was significantly associated with a higher prevalence of frailty after adjusting for potential confounders. In our study, males, individuals aged >60, former alcohol consumers, individuals with hypertension, and individuals with DM were more likely to be in the fourth quartile when it comes to added sugars. Similar results were observed for >25% of calories from added sugars. However, in our study, individuals with low BMI were more likely to get frailty than individuals with high BMI, and we speculated that when the body experiences severe malnutrition, it will be more affected than high BMI individuals, including immune dysfunction and low-stress responses. However, the level of smoking and alcohol intake does not affect the level of added sugar intake. Added sugars will lead to frailty. Overall, it is recommended to limit the intake of added sugars and change unhealthy lifestyle habits to prevent and manage frailty. In this research, the concept of frailty goes beyond chronological age and reveals that as people grow older, they may experience declines in physiological systems, leading to a high risk of adverse health outcomes. Therefore, the FI serves as a significant research tool, enabling healthcare professionals to better identify and understand the characteristics of the frailty population so that tailored interventions may be developed to protect their quality of life and overall wellbeing.

This study has several strengths. First, researchers were recruited directly from the NHANES database, which uses a complex stratified sampling method to enhance the representativeness of our study findings. Second, the standardized questionnaire utilized by NHANES ensures high consistency among participants. However, our study also faces limitations. First, being a cross-sectional study, it cannot establish causality, necessitating further prospective studies to validate our conclusions. Moreover, it is well known that frailty can decrease an individual’s ability to chew hard foods, leading to a preference for softer foods that are higher in sugar content. In this study, the conclusion regarding the association between added sugars and frailty may be a false positive result due to the increased proportion of added sugars in the daily diet of frail individuals. Second, as sugar intake data were obtained from participants’ dietary recall questionnaires, there may be some reporting bias. Third, although we made efforts to control for all potential confounding factors affecting frailty, not all confounders could be eliminated, potentially introducing bias. Finally, as introduced in our introduction, a long-term high intake of added sugars may indeed impair neurotransmitter systems in the brain, consequently affecting mental health. Therefore, the frailty observed in this study could potentially stem from underlying psychological issues rather than genuine physical frailty. It may represent a misperception of one’s own physical abilities due to psychological factors rather than a true state of physical frailty. This aspect adds a certain level of complexity and potential confounding to the conclusions of our study.

## Conclusion

In this cross-sectional study, we found that added sugars were positively associated with the prevalence of frailty. We hope this study can provide some help for the prevention and treatment of frailty. More prospective studies are still needed to further explore the relationship between added sugars and frailty.

## Data availability statement

The datasets presented in this study can be found in online repositories. The names of the repository/repositories and accession number(s) can be found in the article/supplementary material.

## Ethics statement

The study protocol was authorized by National Center for Health Statistics (NCHS) Research Ethics Review Board (Protocol #2005-06, #2011-17, #2018-01).

## Author contributions

JJ: Writing – original draft. JQ: Writing – original draft. YT: Writing – original draft. MX: Writing – original draft. BP: Writing – original draft. CW: Writing – review & editing. GH: Writing – review & editing. DQ: Writing – review & editing.
